# Construction of Designer Selectable Marker Deletions with a CRISPR-Cas9 Toolbox in *Schizosaccharomyces pombe* and New Design of Common Entry Vectors

**DOI:** 10.1534/g3.117.300363

**Published:** 2018-01-10

**Authors:** Yu Zhao, Jef D. Boeke

**Affiliations:** * Institute for Systems Genetics, NYU Langone Health, New York 10016; † Department of Biochemistry and Molecular Pharmacology, NYU Langone Health, New York 10016

**Keywords:** marker deletion, *Schizosaccharomyces pombe*, CRISPR-Cas9 toolbox, new plasmids

## Abstract

Vectors encoding selectable markers have been widely used in yeast to maintain or express exogenous DNA fragments. In the fission yeast *Schizosaccharomyces pombe*, several engineered markers have been reported and widely used, such as *ura4*^+^ and *ScLEU2* from *Saccharomyces cerevisiae*, which complement *ura4* and *leu1* mutations, respectively. These two auxotrophic markers share no homology with the *S. pombe* genome; however, most others can recombine with the genome due to sequence homology shared between the genomic and plasmid-borne copies of the markers. Here, we describe a CRISPR-Cas9 toolbox that can be used to quickly introduce “designer” auxotrophic marker deletions into host strains, including *leu1*-Δ*0*, *his3*-Δ*0*, and *lys9*-Δ*0*. Together with *ura4-D18*, this brings the total number of available designer deletion auxotrophic markers to four. The toolbox consists of a Cas9-gRNA expression vector and a donor DNA plasmid pair for each designer deletion. Using this toolbox, a set of auxotrophic *S. pombe* strains was constructed. Further, we reorganized essential components in the commonly used pREP series of plasmids and assembled the corresponding auxotrophic marker gene onto these plasmids. This toolbox for producing designer deletions, together with the newly developed strains and plasmids, will benefit the whole yeast community.

In fission yeast, a selectable marker or drug resistance gene ensures stable maintenance of plasmids during cell growth. These plasmids encoding marker genes have been widely used in yeast to maintain or express exogenous DNA fragments. Markers from biosynthesis pathways are preferred because of their compatibility with chemically defined culture media and minimal effect on fitness. The first marker gene *ura4^+^* was reported decades ago and the *ura4-D18* deletion can be complemented with plasmids containing the *ura4* marker (Grimm *et al.* 1988). Following that, several general cloning vectors, expression vectors, and integration vectors were constructed using it (Brun *et al.* 1995; Maundrell 1990; Keeney and Boeke 1994). Since then, more auxotrophic markers have been reported, including *leu1*, *his1*, *his3*, *his7*, *arg3*, *ade1*, *lys1*, *lys2*, and *cys2* (Kikuchi *et al.* 1988; Hoffman and Hoffman 2006; Fennessy *et al.* 2014), with many of them cloned and assembled into vectors. Currently, in fission yeast, *ura4*, *his3*, *leu1*, and *ade6* remain the most commonly used markers and are mutated in many popular host strains. Excepting *ura4*, the use of such vectors is limited because the marker genes are mutated in a single base pair, such as *leu1-32* and *ade-M210*, or partially deleted as in *his3-D1*. These mutations can revert (for the point mutations) and/or be repaired via homologous recombination with wild-type marker genes from plasmids, allowing for vector loss. To partially address this problem, *ScLEU2* from *Saccharomyces cerevisiae* (*Sa. cerevisiae*) has been used to complement *leu1-32*, though more optimal choices are desirable, especially when studying the expression and regulation of multiple genes, as well as potential applications in biotechnology and synthetic biology (Ikushima *et al.* 2015; Mitchell *et al.* 2017; Smith *et al.* 2003; Gibson *et al.* 2008, 2010; Annaluru *et al.* 2014). However, creating marker deletions without introducing any new markers or LoxP sites remains complicated and laborious.

In *Sa. cerevisiae*, the standard “designer deletion” strains and CEN/ARS/2 μ pRS plasmids were published 20 yr ago, providing a foundation for budding yeast studies since then (Brachmann *et al.* 1998; Sikorski and Hieter 1989; Christianson *et al.* 1992). In *S. pombe*, the completion of a whole-genome sequence gives us a comprehensive view of biosynthetic processes (Wood *et al.* 2002). Recently, the CRISPR-Cas9 system has been adapted for genome editing in yeast and mammalian cells (Cong *et al.* 2013; DiCarlo *et al.* 2013; Jacobs *et al.* 2014). We sought to use these advanced molecular techniques to introduce standardized tools, similar to those that exist for *Sa. cerevisiae*, into fission yeast.

We therefore describe a toolbox employing CRISPR-Cas9 to easily generate designer deletions of the commonly used markers *leu1* and *his3*. We also introduced another new marker, *lys9*, on chromosome II, which encodes saccharopine dehydrogenase and is required for lysine biosynthesis. Strains generated by our method with *ura4-D18*, *leu1*-Δ*0*, *his3*-Δ*0*, or *lys9*-Δ*0* are auxotrophic for uracil, leucine, histidine, or lysine, respectively. We then cloned the corresponding genes (*ura4*, *leu1*, *his3*, or *lys9*) into a series of new shuttle vectors able to replicate in both *Escherichia coli* and *S. pombe*, similar to the widely used pRS plasmids in *Sa. cerevisiae*. Finally, we describe a set of fission yeast strains constructed using these tools.

## Materials and Methods

### Media

*S. pombe* strains were cultured in rich YES media (Burke and Gould 1994) or chemically defined PMG media supplied with the necessary supplements (Moreno *et al.* 1991). PMG5 media contains all five supplements—adenine, histidine, leucine, uracil, and lysine—at 225 mg/L, and drop-out media was prepared by leaving out one of the five amino acids. Top10 *E. coli* was grown in Luria Broth media. In order to select bacteria with drug-resistant genes, carbenicillin (Sigma-Aldrich) or kanamycin (Sigma-Aldrich) was used at a final concentration of 75 or 50 µg/ml, respectively. Agar was added to 2% for preparation of solid media.

### Plasmids

All plasmids constructed in this study are listed in Table 2 and Supplemental Material, Table S1, Table S2, and Table S3. The original Cas9-sgRNA plasmid pMZ374 was obtained from Addgene (plasmid #59896). Its *Csp*CI placeholder was replaced with a *Not*1 recognition site, generating plasmid pYZ033 (Jacobs *et al.* 2014). The sgRNA cassette can be easily assembled into *Not*1-digested pYZ033 using Gibson assembly (Gibson *et al.* 2009). To facilitate future manipulations, we also constructed Cas9-gRNA-*Not*I entry plasmids with *ScLEU2*, *Spleu1*, *Sphis3*, *Splys9*, and *kanMX* markers. Then, guide RNAs targeting *leu1*, *his3*, and *lys9* were designed and assembled into pYZ033, generating pYZ146, pYZ164, and pYZ173, respectively.

Donor DNA plasmids to delete *leu1*, *his3*, and *lys9* contained ∼1000 bp of upstream and downstream sequence surrounding each marker, and were cloned from wild-type genomic DNA into a vector using the Zero Blunt TOPO Cloning Kit (Invitrogen), then assembled with the empty entry vector pAV10 using yeast Golden Gate (yGG) (Agmon *et al.* 2015), generating plasmids pYZ145, pYZ149, and pYZ172 as the donor DNA to be used with *leu1*, *his3*, and *lys9*, respectively. *Not*I sites were incorporated at both ends of each fragment for easy release.

We also constructed a set of plasmids with the selectable markers described above, and reorganized the essential components to correspond to the pREP1 and pREP2 plasmids (Maundrell 1990). The *nmt1/nmt41/nmt81* promoters and *ars1* replicating region were cloned from original pREP1/pREP41/pREP81 plasmids. An ampicillin resistance cassette (AmpR) and *lacZ*α multiple cloning site (MCS) were cloned from the pRS416 plasmid (Brachmann *et al.* 1998). These fragments were all assembled using yGG. Several synonymous mutations were introduced to eliminate or add several endonuclease recognition sites using multichange isothermal mutagenesis (Mitchell *et al.* 2013), in order to keep MCS restriction sites in the expression plasmids unique (Figure S1).

### Transformation and strain construction

*S. pombe* cells were transformed with 1 μg circular or linearized plasmids using the standard LiOAc transformation protocol (Forsburg and Rhind 2006). To introduce the *leu1* deletion into *ura4-D18* host strains, the Cas9-gRNA plasmid pYZ146 and linearized donor DNA pYZ145 were cotransformed, and selected on PMG5–Ura plates after 5 d of incubation at 30°. Colonies were randomly picked and streaked to fresh PMG5–Ura plates, then screened using colony PCR. Colonies showing the *leu1* deletion were inoculated in liquid YES media to allow for mitotic loss of the plasmid, which was confirmed by replica plating. Other strains with *his3* and *lys9* deletions were constructed using a similar strategy. Strains with all four deletions were constructed through meiotic homologous recombination and tetrad dissections. All *S. pombe* strains constructed in this study are listed in Table 1 and Table S4.

**Table 1:** Yeast strains

Strain	Mating	Genotype					Source
YZY558	*h^+^*	*ura4-D18*	*leu1*-Δ*0*	*his3*-Δ*0*	*lys9*-Δ*0*		This study
YZY559	*h^+^*	*ura4-D18*	*leu1*-Δ*0*	*his3*-Δ*0*	*lys9*-Δ*0*	*ade6-M210*	This study
YZY560	*h^+^*	*ura4-D18*	*leu1*-Δ*0*	*his3*-Δ*0*	*lys9*-Δ*0*	*ade6-M216*	This study
YZY561	*h*^−^	*ura4-D18*	*leu1*-Δ*0*	*his3*-Δ*0*	*lys9*-Δ*0*		This study
YZY562	*h*^−^	*ura4-D18*	*leu1*-Δ*0*	*his3*-Δ*0*	*lys9*-Δ*0*	*ade6-M210*	This study
YZY563	*h*^−^	*ura4-D18*	*leu1*-Δ*0*	*his3*-Δ*0*	*lys9*-Δ*0*	*ade6-M216*	This study
BP231	*h^+^*	*ura4-D18*					Jürg Kohli
BP232	*h*^−^	*ura4-D18*					Jürg Kohli
YZY579	*h^+^*		*leu1*-Δ*0*				This study
YZY580	*h*^−^		*leu1*-Δ*0*				This study
YZY581	*h^+^*			*his3*-Δ*0*			This study
YZY582	*h*^−^			*his3*-Δ*0*			This study
YZY583	*h^+^*				*lys9*-Δ*0*		This study
YZY584	*h*^−^				*lys9*-Δ*0*		This study
YZY585	*h^+^*	*ura4-D18*	*leu1*-Δ*0*				This study
YZY586	*h*^−^	*ura4-D18*	*leu1*-Δ*0*				This study
YZY587	*h^+^*	*ura4-D18*		*his3*-Δ*0*			This study
YZY588	*h*^−^	*ura4-D18*		*his3*-Δ*0*			This study
YZY589	*h^+^*	*ura4-D18*			*lys9*-Δ*0*		This study
YZY590	*h*^−^	*ura4-D18*			*lys9*-Δ*0*		This study
YZY591	*h^+^*		*leu1*-Δ*0*	*his3*-Δ*0*			This study
YZY592	*h*^−^		*leu1*-Δ*0*	*his3*-Δ*0*			This study
YZY593	*h^+^*		*leu1*-Δ*0*		*lys9*-Δ*0*		This study
YZY594	*h*^−^		*leu1*-Δ*0*		*lys9*-Δ*0*		This study
YZY595	*h^+^*			*his3*-Δ*0*	*lys9*-Δ*0*		This study
YZY596	*h*^−^			*his3*-Δ*0*	*lys9*-Δ*0*		This study
YZY597	*h^+^*	*ura4-D18*	*leu1*-Δ*0*	*his3*-Δ*0*			This study
YZY598	*h*^−^	*ura4-D18*	*leu1*-Δ*0*	*his3*-Δ*0*			This study
YZY599	*h^+^*	*ura4-D18*	*leu1*-Δ*0*		*lys9*-Δ*0*		This study
YZY600	*h*^−^	*ura4-D18*	*leu1*-Δ*0*		*lys9*-Δ*0*		This study
YZY601	*h^+^*	*ura4-D18*		*his3*-Δ*0*	*lys9*-Δ*0*		This study
YZY602	*h*^−^	*ura4-D18*		*his3*-Δ*0*	*lys9*-Δ*0*		This study
YZY603	*h^+^*		*leu1*-Δ*0*	*his3*-Δ*0*	*lys9*-Δ*0*		This study
YZY604	*h*^−^		*leu1*-Δ*0*	*his3*-Δ*0*	*lys9*-Δ*0*		This study

### Data availability

The authors state that all data necessary for confirming the conclusions presented in the article are represented fully within the article.

## Results and Discussion

### Design of the S. pombe CRISPR-Cas9 toolbox

Currently, the most commonly used marker in *S. pombe* is *ura4*. The *ura4-D18* deletion is defined by two *Hin*dIII cutting sites on chromosome III, which are located outside the *ura4* promoter and terminator regions without disturbing expression of neighboring genes, as judged by normal growth. The complementing fragment is just 1.7 kb, a conveniently small size, and can be counterselected with 5-fluoro-orotic acid (5-FOA) (Boeke *et al.* 1984). We chose *ura4-D18* as a starting strain, and used *ura4* as the selectable marker for our CRISPR-Cas9 plasmids.

**Figure 1:**
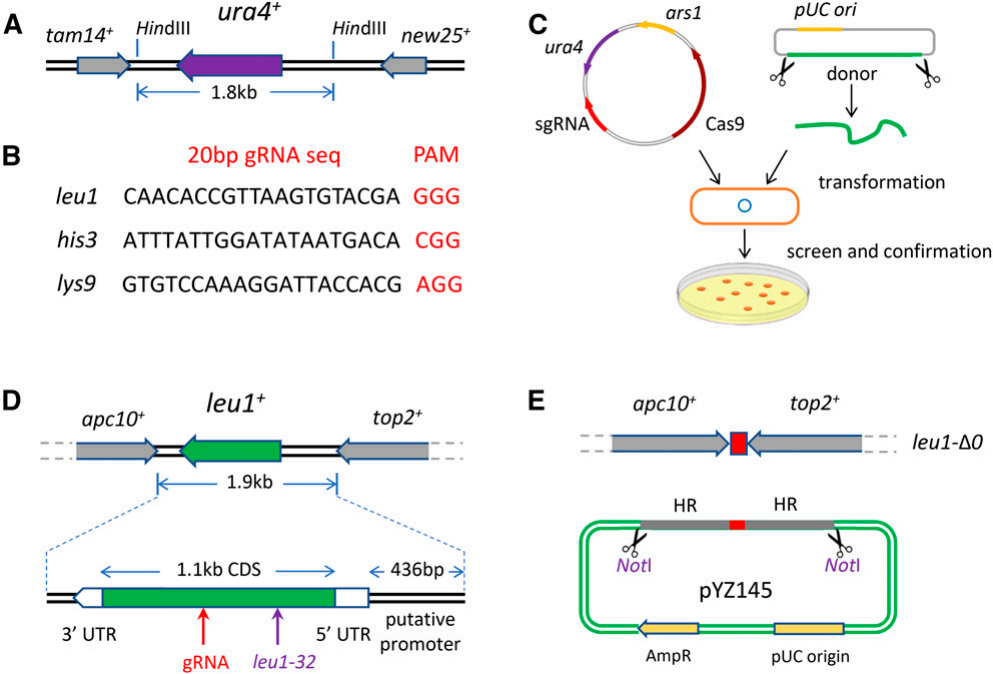
Design of CRISPR-Cas9 toolbox and *leu1*-Δ*0* in *S. pombe*. (A) *ura4-D18* structure. The *ura4^+^* transcript is shown in purple. The *tam14^+^* and *new25^+^* transcripts are shown in gray. (B) For each marker, one 20 bp gRNA sequence was selected, followed by the PAM sequence NGG. (C) Toolbox consists of a Cas9-gRNA expression vector specified for each deletion and another *Not*I-linearized donor DNA. (D) *leu1*-Δ*0* deletion. A 1.9 kb region between the *apc10^+^* and *top2^+^* transcript ends was deleted. The *leu1-32* mutation site was labeled in purple. The gRNA targeting *leu1* was selected from the *leu1^+^* CDS and labeled in red. (E) To minimize the effect on cell growth, one 119-bp AT-rich terminator (Scirica *et al.* 2013) from chromosome I was integrated. This design was achieved through the donor DNA in pYZ145. AmpR, ampicillin resistance; CDS, coding sequence; CRISPR, clustered regularly interspaced short palindromic repeats; gRNA, guide RNA; HR, homologous recombination; PAM, protospacer adjacent motif; UTR, untranslated region.

This toolbox consists of one Cas9-gRNA plasmid expressing the active Cas9 enzyme and sgRNA, and one plasmid that can be linearized to provide the donor DNA for each designer deletion (Figure 1C). We have included *leu1*-Δ*0*, *his3*-Δ*0*, and *lys9*-Δ*0*; however, more markers could be easily introduced following the same principle. To use this toolbox, one need only cotransform the two components into yeast cells and screen for positive colonies by PCR, followed by phenotypic testing using replica plating. The Cas9-gRNA plasmid can be easily lost by incubating the strain in nonselective YES medium overnight, followed by replica plating to confirm plasmid loss.

Two important criteria governed our choice of genes for designer deletions. First, markers used heavily in previous work were chosen over compatriots in the same pathway (*e.g.*, *leu1* was preferred over other *leu* genes). Secondly, we avoided markers that had nearby essential genes that might use overlapping promoter elements. We aimed to delete the marker of interest as completely as possible, without affecting the expression of flanking genes, and especially not those flanking genes that are required for fast growth. The idea is to maximize the utility of the marker, minimize the homology with the marker used on the plasmid vector, and maximize the overall fitness of the auxotrophic strain.

### Complete deletion of leu1 and design of the donor DNA for leu1-Δ0

One commonly used marker is *leu1* on chromosome II, where the mutation *leu1-32* can be complemented with *ScLEU2* (Kikuchi *et al.* 1988). To maximize compatibility with strains and plasmids currently in use, we selected *leu1*-Δ*0* for inclusion. The gRNA sequence was selected from +702 to +721 in the CDS region, which can be used to target both *leu1^+^* and *leu1-32* host strains (Figure 1D). The genes downstream (*apc10^+^*) and upstream (*top2^+^*) are both essential, but their transcripts do not overlap with *leu1*. We selected a deletion region of 1.9 kb, containing the full length of *leu1* CDS and untranslated regions (UTRs) (Figure 1D).

The donor DNA, with flanking homologous regions of 1 kb in length at each end, was provided from plasmid pYZ145 linearized with *Not*I digestion. The 119 bp AT-rich region between *rpl301^+^* and *mde10^+^*, which are similarly organized tail-to-tail (genome coordinates: 1757331–1757559), was integrated as a convergent terminator element, in order to maintain the expression of *apc10^+^* and *top2^+^* (Figure 1E). This element is kept short, in order to minimize the likelihood of potentially deleterious recombination with the native region on chromosome I.

### Complete deletion of his3 and design of donor DNA for his3-Δ0

Another popular selectable marker is *his3*, and its partial deletion *his3-D1* has been widely used in host strains (Burke and Gould 1994). In *his3-D1*, most of the CDS (+19 to +1144) was deleted, but the promoter, UTR, and terminator elements remained in place, providing potential opportunities for gene conversion if a *his3^+^* allele is included in a plasmid. A complete deletion removing all homology is preferable.

**Figure 2:**
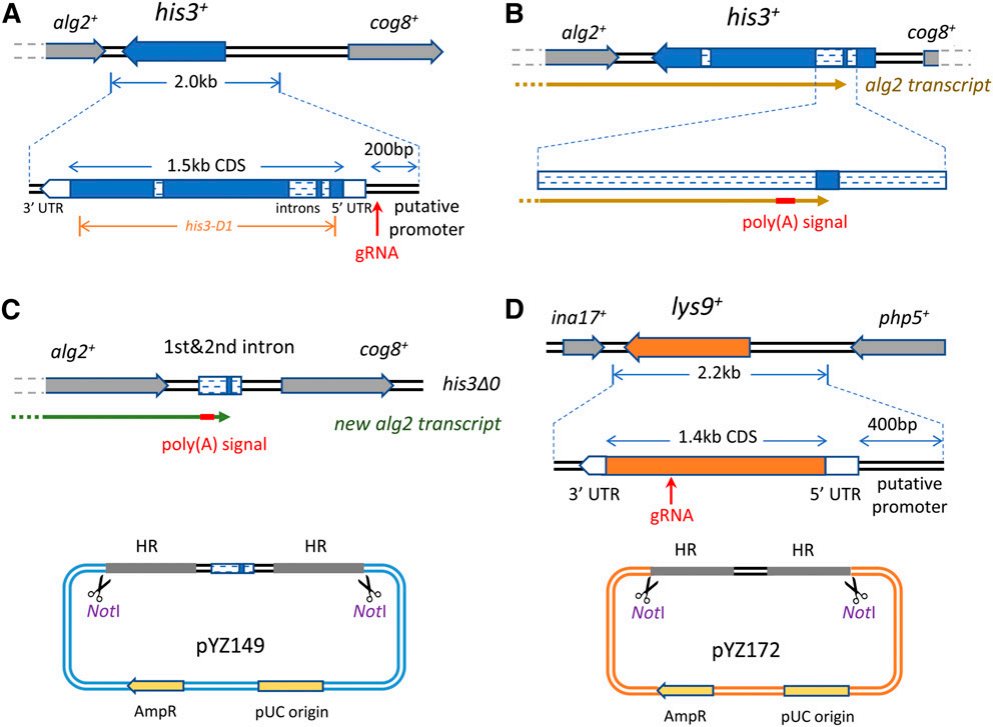
Design of deletions and donor DNAs for *his3*-Δ*0* and *lys9*-Δ*0*. (A) A 2.0 kb region containing the *his3^+^* CDS was deleted, generating *his3*-Δ*0*. The gRNA was selected upstream of the CDS in order to make the toolbox compatible with existing *his3-D1* strains. The region 200 bp upstream of the 5′ UTR was arbitrarily defined as the putative promoter in the plasmid. (B) The *his3^+^* gene has three introns and four exons. The convergently transcribed downstream gene *alg2^+^* is essential, and its transcript ends in the second exon of *his3^+^*. The poly(A) signal located at the second intron, and the first intron, may work as the terminator for *alg2^+^*. (C) The region from the first to second introns was kept in the genome for *his3*-Δ*0*, in order to minimize any possible effects on *alg2^+^* transcripts. Since all the introns were removed when *his3* was used as the marker, this design leaves no significant homologous region with the plasmid. The donor DNA was the *Not*I digestion product from the pYZ149 plasmid, and a 1 kb homologous region was included on each side for integration. (D) *lys9* is a new selectable marker. The *lys9*-Δ*0* strain is auxotrophic for lysine and the deletion has no detectable effect on cell growth. A 2.2 kb region was deleted, including the 1.6 kb transcription unit, and the 400 bp upstream and 150 bp downstream regions. The gRNA targets the CDS. Donor DNA is a *Not*I digest of pYZ172. AmpR, ampicillin resistance; CDS, coding sequence; gRNA, guide RNA; HR, homologous recombination; UTR, untranslated region.

The *his3^+^* gene has three introns and four exons, and its flanking genes *alg2^+^* and *cog8^+^* are both essential (Figure 2A). The gRNA sequence was selected from the putative promoter region in order to allow the conversion of existing *his3-D1* strains. But more challengingly, although the CDS regions do not overlap, the *alg2* transcript end is nested within the exceedingly short second exon of *his3*, which is just 12 bp in length (Figure 2B). The *his3-D1* deletion has no detectable effect on cell growth. However, the *alg2* polyadenylation signal sequence (5′-AAUAAA-3′) for post-transcriptional modification is located near the transcript end, and maps to the second intron of *his3*. The first intron is AT-rich and likely functions as the site of termination for *alg2* (Figure 2B). To minimize any problems related to 3′ end formation, we retained the region from the first intron through the second intron in the donor DNA so that in *his3*-Δ*0* strains, the 3′ mRNA processing and transcriptional termination of *alg2* remain in their normal configuration (Figure 2C). Since all introns were removed from the *his3* CDS used in plasmids, only the 12 bp exon sequence in the middle remains homologous to the genomic sequence.

For the downstream essential gene *cog8*, one 496 bp region upstream of its transcript was kept in the genome as the putative promoter. The 200 bp region upstream of the *his3* transcript was deleted from the genome, and this sequence was retained as the *his3* promoter in plasmids, which was previously shown in pBG1 to sufficiently complement *his3-D1* (Burke and Gould 1994).

The Cas9-gRNA plasmid for *his3*-Δ*0* was pYZ164. The donor DNA was obtained from plasmid pYZ149 linearized with *Not*I digestion (Figure 2C).

### Complete deletion of lys9 and design of donor DNA for lys9-Δ0

In addition to *ura4-D18*, *leu1*-Δ*0*, and *his3*-Δ*0*, we also introduced a fourth selectable marker, *lys9*. Lysine is used as one of the five supplements in YES rich media and PMG5 defined media. *lys1^+^* and *lys2^+^* from the lysine biosynthesis pathway have previously been cloned and reported to work as potential selectable markers (Chino *et al.* 2010; Hoffman and Hoffman 2006). Here, we used *lys9* as the marker for two reasons. First, its CDS is only 1.4 kb in length, whereas *lys1* and *lys2* CDS are 4.2 and 2.2 kb in length, respectively. This compact size makes *lys9* preferable for use in plasmids. Second, the genomic context of *lys9* is simple. The downstream gene *ina17^+^* and upstream gene *php5^+^* are both nonessential, and the deletion is likely to have minimal effect on their expression because of their convergent transcription pattern. The gRNA that we designed targets the *lys9* CDS region (plasmid pYZ173), and our donor DNA was released with *Not*I digestion from pYZ172 (Figure 2D).

### Strain construction using the CRISPR-Cas9 toolbox

After the toolbox was successfully completed, we tested it and constructed a set of *S. pombe* strains with designed deletions (Table 1). We first introduced *leu1*-Δ*0* and generated a new strain YZY477 (*h*^90^*ura4-D18 leu1*-Δ*0 his3-D1 ade6-M210*). After 5 d of incubation on PMG5–Ura plates, we observed yeast colonies of a variety of sizes. Around 24 colonies were randomly picked and screened for *leu1*-Δ*0* by colony PCR. Deletion efficiency was 10–20% and all positive colonies were relatively small in size. This was consistent with a previous report of Cas9 toxicity in fission yeast (Rodríguez-López *et al.* 2017). We hypothesized that slower growth was caused by Cas9 protein toxicity, as bigger colonies turned out to be negative for the deletion, possibly because Cas9 was inactive. Following a similar strategy, we continued by introducing *leu1*-Δ*0*, *his3*-Δ*0*, and *lys9*-Δ*0* deletions, generating YZY540 (*h*^−^ *ura4-D18 leu1*-Δ*0 lys9*-Δ*0*), YZY541 (*h*^−^ *ura4-D18 leu1*-Δ*0 lys9*-Δ*0 ade6-M216*) and YZY543 (*h^+^ ura4-D18 leu1*-Δ*0 his3*-Δ*0 ade-M210*).

By crossing YZY540 (*h*^−^) and YZY543 (*h^+^*) or YZY541 (*h*^−^) and YZY543 (*h^+^*), we generated final strains containing all four designer deletions, with *ade6^+^*, *ade6-M210*, or *ade6-M216* for both mating types (Table 1). We retained *ade6-M210* in order to use that to complement *ade6-M216* and keep the diploid status. In fission yeast, *leu1*, *his3*, *lys9*, and the mating-type region are all located on chromosome II, whereas *ura4* and *ade6* are located on chromosome III. Compared to the introduction of new designer deletions, repair of deletions back to wild-type is much easier. One can generate many more combinations by transformation with PCR products of wild-type marker genes and selection of prototrophic colonies, or by backcrossing to the wild-type (Table 1).

**Figure 3:**
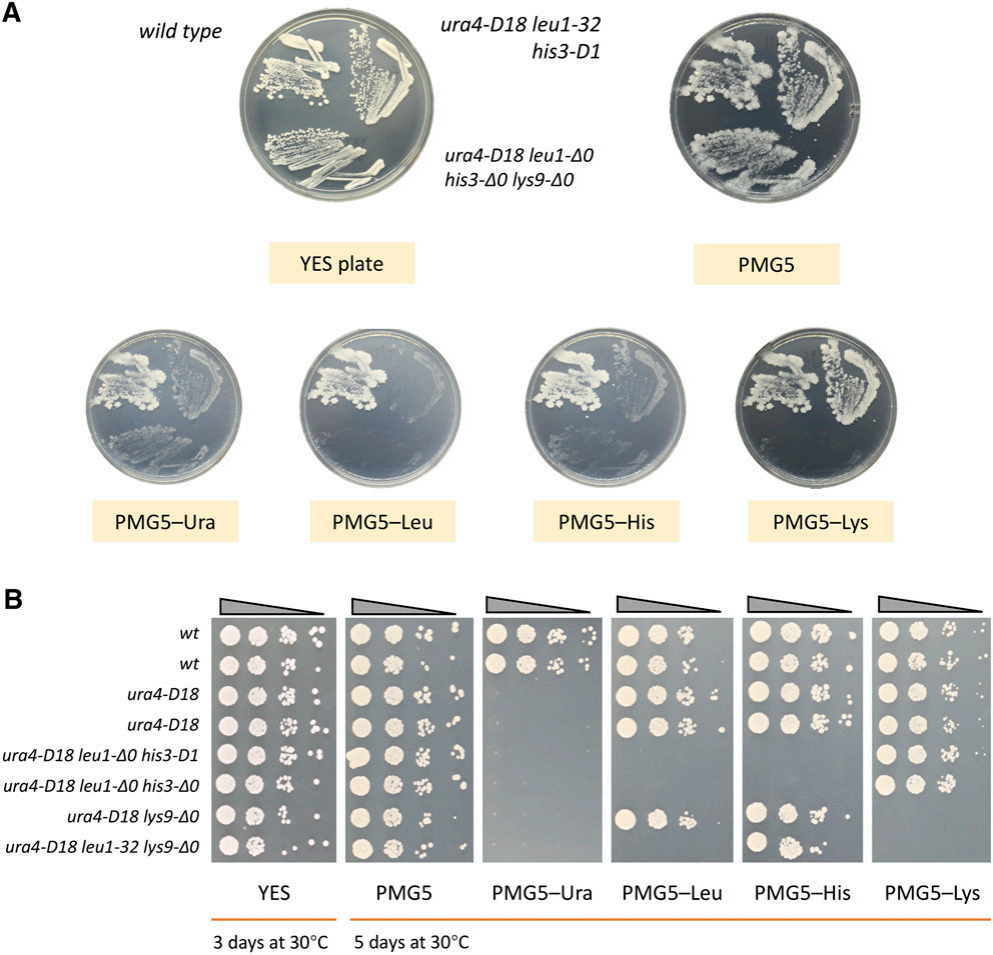
Deletion confirmation with replica plates and strain fitness assay. (A) From single colonies, we streaked the marker deletion strains on the YES plate. Then, the plate was replica plated to a PMG5 plate with all five supplements, and the indicated drop-out plates. (B) Fitness testing. Dots represent 10-fold dilutions left to right. Compared to wild-type or *ura4-D18* strains, the new deletions caused no detectable growth defect. PMG5, *S. pombe* minimal glutamate medium with 5 supplements; YES, yeast extract with supplements.

After the Cas9 plasmid was lost, strain phenotypes were confirmed by replica plating from YES to PMG5 and drop-out media (Figure 3A). We also checked strain fitness using single colony formation assays. All deletion strains grew normally on rich and PMG5 media compared to the wild-type strains (Figure 3B).

### New plasmids for general purpose and protein expression in S. pombe

Many cloning vectors for *S. pombe* have been constructed using different selectable markers, especially *ura4* and *ScLEU2*, complementing *ura4-D18* and *leu1-32*, respectively. The majority of them can be divided into two categories: general purpose vectors and expression vectors. Both vector types have common essential components including *ars1* for replication in *S. pombe*, drug resistance genes such as AmpR or KanR, and a pUC/ColE1 *E. coli* replication origin. Two general purpose vectors, pSP1 and pSP2, were constructed 25 yr ago, with *ScURA3* and *ScLEU2* as the markers, respectively (Cottarel *et al.* 1993). For expression vectors, the *nmt* promoter, which becomes active in the media without thiamine, is the most commonly used to control expression in fission yeast. The pREP1 or pREP2 plasmids were constructed ∼30 yr ago with *ScLEU2* or *ura4* as markers, respectively (Maundrell 1990, 1993). The markers were inserted into the *lacZ*α MCS in those vectors, blocking use of the blue–white screening feature. It was also not easy to replace current markers with new ones developed in this study (Table 2).

**Table 2:** Plasmids

Plasmid*^a^*	Antibiotic	Marker	Description	Addgene ID
pYZ033	AmpR	*Sp ura4*	Entry vector for *S. pombe* CRISPR-Cas9 system.	98404
pYZ292	AmpR	*Sc LEU2*	Entry vector for *S. pombe* CRISPR-Cas9 system.	102686
pYZ293	AmpR	*Sp leu1*	Entry vector for *S. pombe* CRISPR-Cas9 system.	102687
pYZ294	AmpR	*kanMX*	Entry vector for *S. pombe* CRISPR-Cas9 system.	102688
pYZ300	AmpR	*Sp his3*	Entry vector for *S. pombe* CRISPR-Cas9 system.	102689
pYZ301	AmpR	*Sp lys9*	Entry vector for *S. pombe* CRISPR-Cas9 system.	102690
pYZ145	AmpR	*—*	Donor DNA for *leu1* deletion in *S. pombe*	98405
pYZ146	AmpR	*Sp ura4*	Cas9-gRNA plasmid for *leu1*	98406
pYZ149	AmpR	*—*	Donor DNA for *his3* deletion in *S. pombe*	98407
pYZ164	AmpR	*Sp ura4*	Cas9-gRNA plasmid for *his3*	98408
pYZ172	AmpR	*—*	Donor DNA for *lys9* deletion in *S. pombe*	98409
pYZ173	AmpR	*Sp ura4*	Cas9-gRNA plasmid for *lys9*	98410
pYZ155	AmpR	*Sp ura4*	nmt1 promoter: expression vector in *S. pombe*	98411
pYZ156	AmpR	*Sp ura4*	nmt41 promoter: expression vector in *S. pombe*	98412
pYZ157	AmpR	*Sp ura4*	nmt81 promoter: expression vector in *S. pombe*	98413
pYZ190	AmpR	*Sp ura4*	General purpose vector in *S. pombe*	98428

ID, identifier; AmpR, ampicillin; CRISPR, clustered regularly interspaced short palindromic repeats; gRNA, guide RNA.

a Only the newly designed plasmids with the *Sp ura4* marker are shown here. Additional plasmids with *Bsa*I-pad, *leu1*, *his3*, and *lys9* are described in Table S2.

In *Sa. cerevisiae*, pRS plasmids, which feature extensive MCSs supporting blue–white sceening and a consistent backbone structure, have become standard entry vectors and backbones for module construction (Brachmann *et al.* 1998; Sikorski and Hieter 1989; Christianson *et al.* 1992; Chee and Haase 2012). We believe that one standard and simple plasmid design with minimal length and high compatibility will benefit the whole *S. pombe* community. As more new marker deletions are introduced, new plasmids are also desirable.

**Figure 4:**
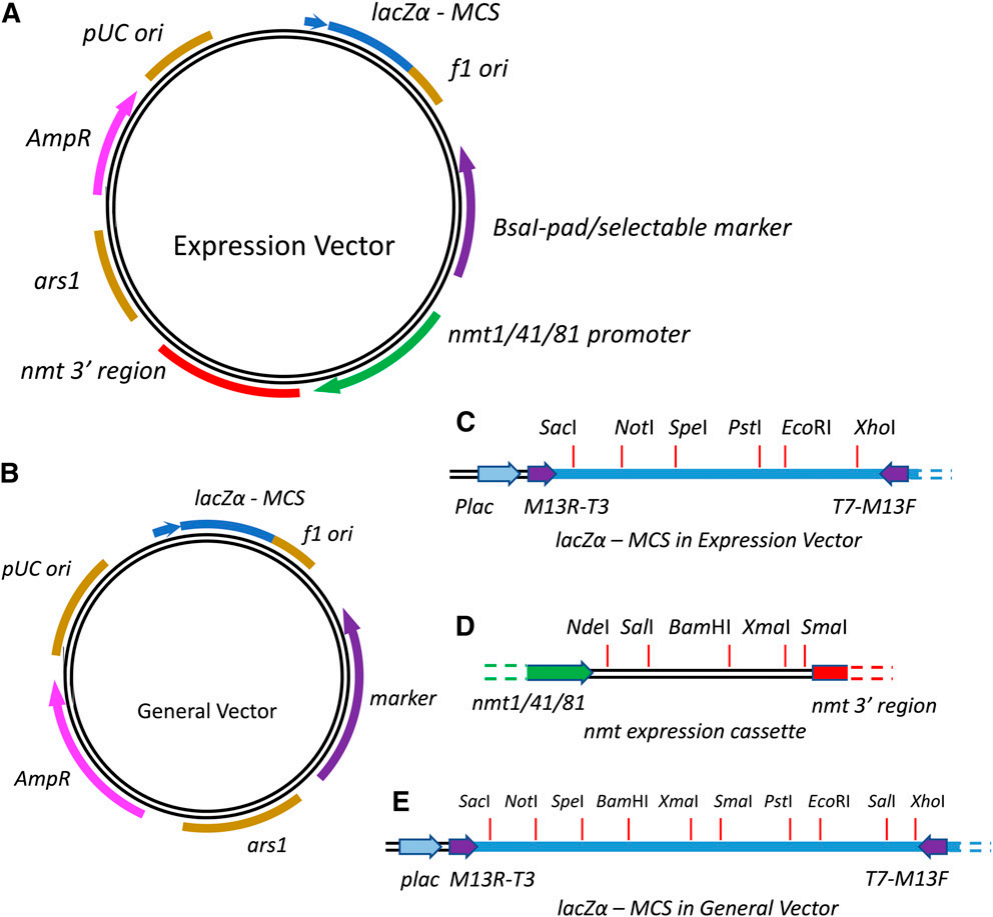
New vector designs for expression and general-purpose vectors. They all have four basic components: (1) an ampicillin resistance gene (AmpR), (2) an *ars1* replication site for *S. pombe*, (3) a pUC/ColE1 replication region for *E. coli*, and (4) the *lacZ*α multiple cloning site. (A) The expression vector diagram. In addition to the four basic parts, the empty *Bsa*I-pad or one selectable marker is included. The marker gene contains several synonymous mutations to eliminate the restriction cutting sites. (B) The vector diagram for general purpose, *e.g.*, complementation cloning. The *nmt* expression cassette was removed to minimize plasmid size. (C) In the *lacZ*α multiple cloning site (MCS) of expression vectors, several unique cutting sites are available. This region is compatible with blue–white screening and can be sequenced with universal primers. (D) Five unique restriction enzyme cutting sites are designed in a second multiple cloning site downstream of the *nmt* promoter, which is consistent with the design of the pREP1/2 plasmid. These sites have been eliminated from the *lacZ*α MCS region through synonymous mutations. (E) In the general vectors, original *lacZ*α was used here with more unique restriction enzyme cutting sites. All sites indicated were checked for uniqueness.

Here, we describe a set of plasmids for general purpose or gene expression at different levels (Figure 4A). At first, we assembled all the necessary components with the “*Bsa*I-pad,” a single 42 bp region containing two *Bsa*I cutting sites, generating the plasmids pYZ182, pYZ183, and pYZ184 with *nmt1*, *nmt41*, and *nmt81* cassettes, respectively. Using this design, marker genes can be easily integrated using yGG or general subcloning. Here, *ura4*, *leu1*, *his3*, and *lys9* were integrated separately. Several synonymous mutations were introduced in order to eliminate unwanted restriction enzyme recognition sites (Figure S1). After the plasmids were transformed in yeast, they were fully functional and successfully complemented *ura4-D18*, *leu1*-Δ*0*, *his3*-Δ*0*, and *lys9*-Δ*0* (Figure S2).

In addition, all plasmids contain a single, consistent *lacZ*α MCS bearing unique restriction sites for many commonly used “six-base” restriction enzymes (Figure 4C). This region is compatible with blue–white screening in the presence of X-gal, and the clone can be sequenced using universal primers such as M13, T7, and T3. Downstream of the *nmt* promoter, several unique cutting sites are also included, which are compatible with pREP series plasmids (Figure 4D). All plasmids constructed in this study have been submitted to Addgene.

### Conclusions

The fission yeast *S. pombe* is a widely used unicellular eukaryotic system, especially for investigating the cell cycle, DNA replication, centromere structure, and chromatin regulation (Beach *et al.* 1982; Sabatinos and Forsburg 2015; Dong *et al.* 2016). *S. pombe* shares several key chromosome features with mammalian cells and humans, making it a useful model for disease and possibly drug discovery. Similar to the budding yeast *Sa. cerevisiae*, *S. pombe* is easy to culture in the laboratory and readily undergoes homologous recombination. Yeast cells are also very biologically safe, since they were originally isolated from fruits or natural fermented beverages. As a result, *S. pombe* is a promising platform for synthetic biology, biotechnology, and industrial fermentation.

Our CRISPR-Cas9 toolbox and newly designed plasmids provide a standardized toolkit to enable these possibilities. Designer deletion of marker genes in host genomes eliminates potential plasmid loss. This brings us more choices for gene expression via plasmids or genome integration using selectable auxotrophic makers. Our new system can be easily applied and is highly compatible with extant designs.

## Supplementary Material

Supplemental material is available online at www.g3journal.org/lookup/suppl/doi:10.1534/g3.117.300363/-/DC1.
