# Supplementary material for: Construction of Designer Selectable Marker Deletions with a CRISPR-Cas9 Toolbox in Schizosaccharomyces pombe and New Design of Common Entry Vectors
Source: G3 (Bethesda). 2018 Jan 10;8(3):789–96. doi: 10.1534/g3.117.300363 (PMC5844300; doi:10.1534/g3.117.300363)
Supplement: Supplementary file 1 [file 789FigureS1.pdf]

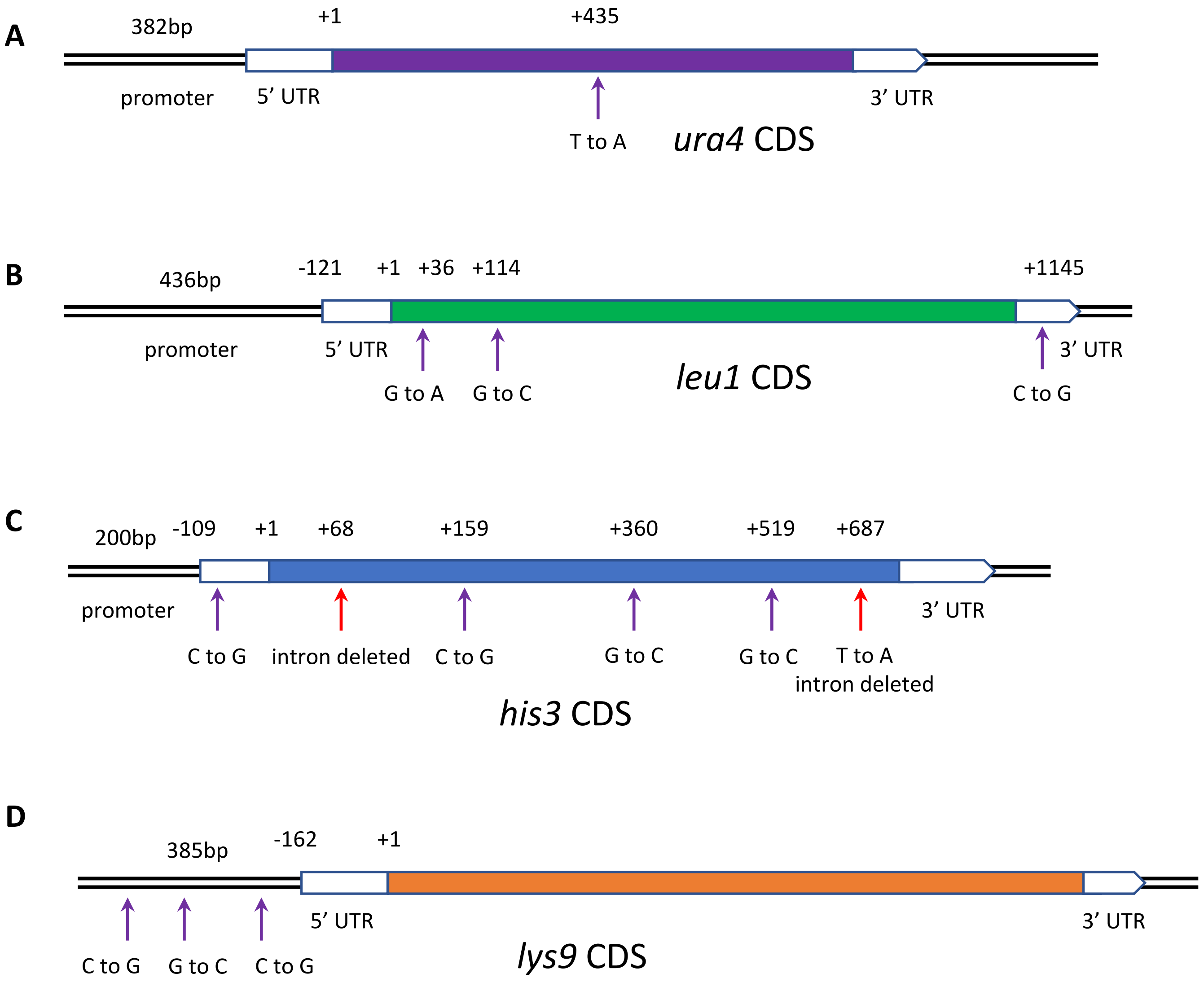

Figure S1 Maker genes used in the plasmids to complement deletions in the genome. (A) To complement *ura4-D18*, one 1764bp region was used in the plasmid, which is the same region used in pREP2 plasmid. One synonymous mutation was introduced in the CDS region, to eliminate the *Bsa*I recognition site. (B) To complement *leu1-Δ0*, one 1895bp region was used in the plasmid. Two synonymous mutations were introduced in the CDS with another one in the 3’ UTR region, to eliminate the recognition sites of *Bsa*I, *Xho*I and *Nde*I, respectively from left to right. (C) To complement *his3-Δ0* or *his3-D1*, one 1707bp region containing *his3* CDS was included in the plasmid. One synonymous mutation was introduced in the 5’UTR region with another four in the CDS region, to eliminate the recognition sites of *Nde*I, *Bam*HI, *Bsa*I, *Xho*I and *Eco*RI. All three introns were removed. (D) To complement *lys9-Δ0*, one 2183bp region containing *lys9* CDS was used in the plasmid. Three synonymous mutations were introduced in the 5’ UTR region, to eliminate the recognition sites of *Eco*RI, *Pst*I and *Eco*RI, respectively.
