# Supplementary figures and images for: Construction of Designer Selectable Marker Deletions with a CRISPR-Cas9 Toolbox in Schizosaccharomyces pombe and New Design of Common Entry Vectors
Source: G3 (Bethesda). 2018 Jan 10;8(3):789–96. doi: 10.1534/g3.117.300363 (PMC5844300; doi:10.1534/g3.117.300363)

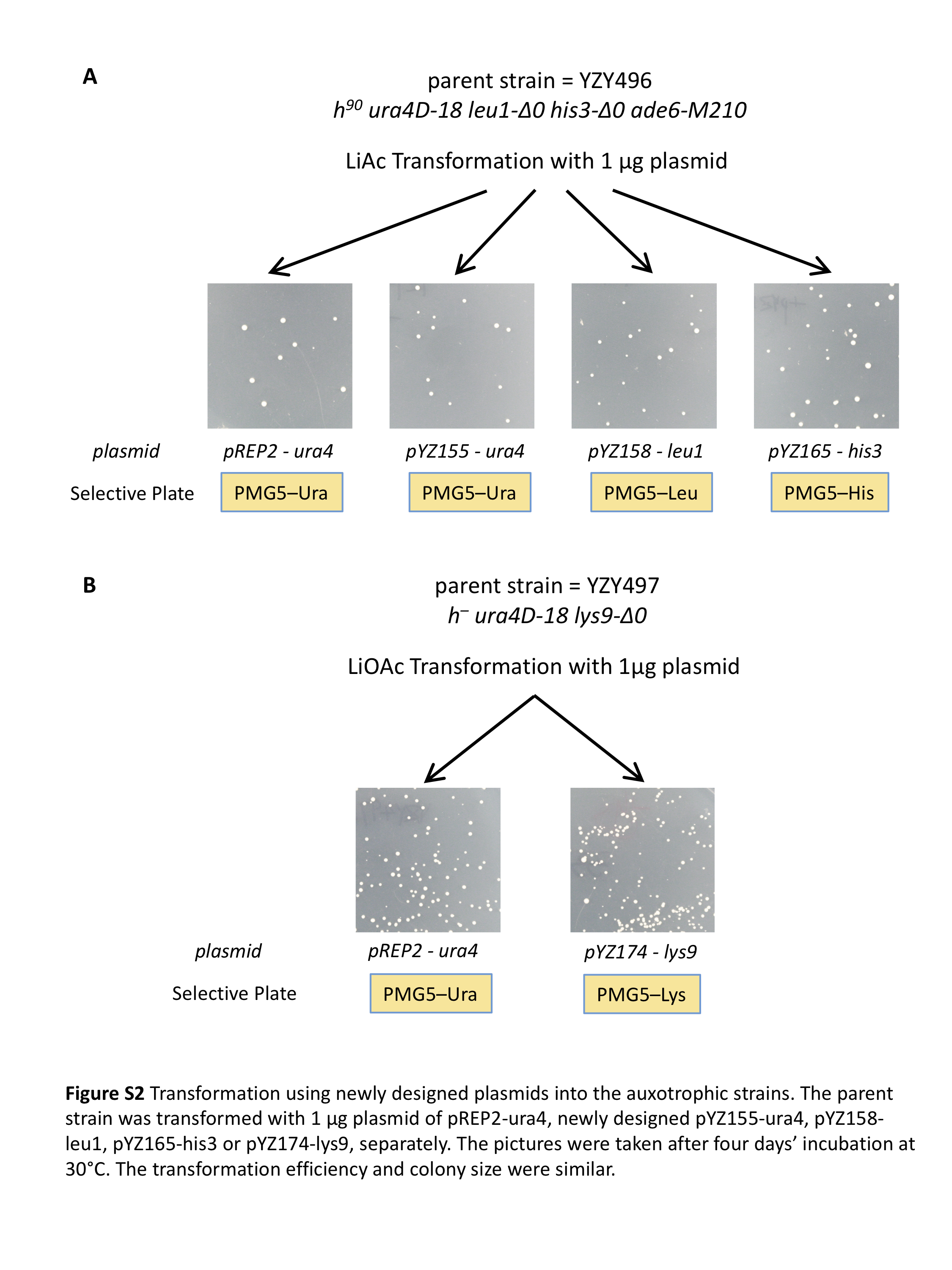

Supplement: Supplementary file 2 [file 789FigureS2.jpg]
