# Supplementary material for: Construction of Designer Selectable Marker Deletions with a CRISPR-Cas9 Toolbox in Schizosaccharomyces pombe and New Design of Common Entry Vectors
Source: G3 (Bethesda). 2018 Jan 10;8(3):789–96. doi: 10.1534/g3.117.300363 (PMC5844300; doi:10.1534/g3.117.300363)
Supplement: Supplementary file 3 [file 789TableS1.docx]

**Table S1. Newly constructed plasmid to introduce the selectable marker deletions in *S. pombe*.**

| **Plasmid** | **Antibiotic** | **Description** | **Addgene ID** |
| --- | --- | --- | --- |
| pYZ145 | AmpR | Donor DNA to introduce *leu1-Δ0* | 98405 |
| pYZ146 | AmpR | CRISPR/Cas9 plasmids with gRNA-Spleu1 | 98406 |
| pYZ149 | AmpR | Donor DNA to introduce *his3-Δ0* | 98407 |
| pYZ164 | AmpR | CRISPR/Cas9 plasmids with gRNA-Sphis3 | 98408 |
| pYZ172 | AmpR | Donor DNA to introduce *lys9-Δ0* | 98409 |
| pYZ173 | AmpR | CRISPR/Cas9 plasmids with gRNA-Splys9 | 98410 |
