# Supplementary material for: Construction of Designer Selectable Marker Deletions with a CRISPR-Cas9 Toolbox in Schizosaccharomyces pombe and New Design of Common Entry Vectors
Source: G3 (Bethesda). 2018 Jan 10;8(3):789–96. doi: 10.1534/g3.117.300363 (PMC5844300; doi:10.1534/g3.117.300363)
Supplement: Supplementary file 4 [file 789TableS2.docx]

**Table S2. Plasmids newly designed and constructed in this study for general purpose or expression in *S. pombe*.**

| **Plasmid** | **Antibiotic** | **Marker** | **Description** | **Addgene ID** |
| --- | --- | --- | --- | --- |
| pYZ155 | AmpR | *Sp ura4* | nmt1 promoter – expression vector in *S. pombe* | 98411 |
| pYZ156 | AmpR | *Sp ura4* | nmt41 promoter – expression vector in *S. pombe* | 98412 |
| pYZ157 | AmpR | *Sp ura4* | nmt81 promoter – expression vector in *S. pombe* | 98413 |
| pYZ158 | AmpR | *Sp leu1* | nmt1 promoter – expression vector in *S. pombe* | 98414 |
| pYZ159 | AmpR | *Sp leu1* | nmt41 promoter – expression vector in *S. pombe* | 98415 |
| pYZ160 | AmpR | *Sp leu1* | nmt81 promoter – expression vector in *S. pombe* | 98416 |
| pYZ165 | AmpR | *Sp his3* | nmt1 promoter – expression vector in *S. pombe* | 98418 |
| pYZ166 | AmpR | *Sp his3* | nmt41 promoter – expression vector in *S. pombe* | 98419 |
| pYZ167 | AmpR | *Sp his3* | nmt81 promoter – expression vector in *S. pombe* | 98420 |
| pYZ174 | AmpR | *Sp lys9* | nmt1 promoter – expression vector in *S. pombe* | 98421 |
| pYZ175 | AmpR | *Sp lys9* | nmt41 promoter – expression vector in *S. pombe* | 98422 |
| pYZ176 | AmpR | *Sp lys9* | nmt81 promoter – expression vector in *S. pombe* | 98423 |
| pYZ182 | AmpR | *-* | nmt1 promoter – expression vector in *S. pombe* with empty BsaI-pad for new markers | 98424 |
| pYZ183 | AmpR | *-* | nmt41 promoter – expression vector in *S. pombe* with empty BsaI-pad for new markers | 98425 |
| pYZ184 | AmpR | *-* | nmt81 promoter – expression vector in *S. pombe* with empty BsaI-pad for new markers | 98426 |
| pYZ189 | AmpR | *-* | general purpose vector in *S. pombe* with empty BsaI-pad | 98427 |
| pYZ190 | AmpR | *Sp ura4* | general purpose vector in *S. pombe* | 98428 |
| pYZ191 | AmpR | *Sp leu1* | general purpose vector in *S. pombe* | 98429 |
| pYZ192 | AmpR | *Sp his3* | general purpose vector in *S. pombe* | 98430 |
| pYZ193 | AmpR | *Sp lys9* | general purpose vector in *S. pombe* | 98431 |
