# Supplementary material for: Construction of Designer Selectable Marker Deletions with a CRISPR-Cas9 Toolbox in Schizosaccharomyces pombe and New Design of Common Entry Vectors
Source: G3 (Bethesda). 2018 Jan 10;8(3):789–96. doi: 10.1534/g3.117.300363 (PMC5844300; doi:10.1534/g3.117.300363)
Supplement: Supplementary file 5 [file 789TableS3.docx]

**Table S3. Newly designed CRISPR/Cas9 plasmids used in *S. pombe*.**

| **Plasmid** | **Antibiotic** | **Marker** | **Description** | **Addgene ID** |
| --- | --- | --- | --- | --- |
| pYZ033 | AmpR | *Sp ura4* | gRNA entry vector with Cas9 | 98404 |
| pYZ292 | AmpR | *ScLEU2* | gRNA entry vector with Cas9 | 102686 |
| pYZ293 | AmpR | *Sp leu1* | gRNA entry vector with Cas9 | 102687 |
| pYZ294 | AmpR | *kanMX* | gRNA entry vector with Cas9 | 102688 |
| pYZ300 | AmpR | *Sp his3* | gRNA entry vector with Cas9 | 102689 |
| pYZ301 | AmpR | *Sp lys9* | gRNA entry vector with Cas9 | 102690 |
