# Supplementary material for: Construction of Designer Selectable Marker Deletions with a CRISPR-Cas9 Toolbox in Schizosaccharomyces pombe and New Design of Common Entry Vectors
Source: G3 (Bethesda). 2018 Jan 10;8(3):789–96. doi: 10.1534/g3.117.300363 (PMC5844300; doi:10.1534/g3.117.300363)
Supplement: Supplementary file 6 [file 789TableS4.docx]

**Table S4. Additional yeast strains**

| **Strain** | **Mating** | **Genotype** | | | | | **Source** |
| --- | --- | --- | --- | --- | --- | --- | --- |
| YZY477 | *h^90^* | *ura4-D18* | *leu1-Δ0* | *his3-D1* |  | *ade6-M210* | This Study |
| YZY496 | *h^90^* | *ura4-D18* | *leu1-Δ0* | *his3-Δ0* |  | *ade6-M210* | This Study |
| YZY498 | *h ^–^* | *ura4-D18* | *leu1-32* |  | *lys9-Δ0* | *ade6-M216* | This Study |
| YZY526 | *h^90^* | *ura4-D18* | *leu1-Δ0* | *his3-Δ0* | *lys9-Δ0* | *ade6-M210* | This Study |
| YZY539 | *h^+^* | *ura4-D18* | *leu1-Δ0* | *his3-D1* |  | *ade-M210* | This Study |
| YZY541 | *h^–^* | *ura4-D18* | *leu1-Δ0* |  | *lys9-Δ0* | *ade6-M216* | This Study |
| YZY543 | *h^+^* | *ura4-D18* | *leu1-Δ0* | *his3-Δ0* |  | *ade6-M210* | This Study |
| BP1101 | *h^+^* | *ura4-D18* | *leu1-32* |  |  | *ade6-M216* | Lab Stock |
| BP1102 | *h^–^* | *ura4-D18* | *leu1-32* |  |  | *ade6-M216* | Lab Stock |
| YZY062 | *h^+^* | *ura4-D18* | *leu1-32* | *his3-D1* |  | *ade6-M210* | Fei Li Lab |
